# Settlement and post-settlement survival rates of the white seabream (*Diplodus sargus*) in the western Mediterranean Sea

**DOI:** 10.1371/journal.pone.0190278

**Published:** 2018-01-11

**Authors:** Amalia Cuadros, Gotzon Basterretxea, Luis Cardona, Adrien Cheminée, Manuel Hidalgo, Joan Moranta

**Affiliations:** 1 Instituto Español de Oceanografía (IEO), Estació d’Investigació Jaume Ferrer, Maó, Spain; 2 Université de Perpignan Via Domitia, CNRS, Centre de Formation et de Recherche sur les Environnements Méditerranéens (CEFREM), UMR 5110, Avenue P. Alduy, Perpignan, France; 3 Department of Ecology and Hydrology, Marine Ecology and Conservation Research Group. University of Murcia, Campus Espinardo, Murcia, Spain; 4 Instituto Mediterráneo de Estudios Avanzados (IMEDEA; UIB-CSIC), Miquel Marqu es 21, Esporles, Mallorca, Spain; 5 IRBio, Department of Animal Biology, Faculty of Biology, University of Barcelona, Avinguda Diagonal, Barcelona, Spain; 6 Septentrion Environnement, Port des Goudes, Traverse Paul, Marseille, France; 7 Instituto Español de Oceanografía (IEO), Centre Oceanogràfic de les Balears (COB), Ecosystem Oceanography Group (GRECO), Moll de Ponent s/n, Palma de Mallorca, Spain; University of Padova, ITALY

## Abstract

Survival during the settlement window is a limiting variable for recruitment. The survival is believed to be strongly determined by biological interactions and sea conditions, however it has been poorly investigated. We examined the settlement patterns related to relevant biotic and abiotic factors (i.e. Density-dependence, wind stress, wave height and coastal current velocity) potentially determining post-settler survival rates of a coastal necto-benthic fish of wide distribution in the Mediterranean and eastern Atlantic, the white seabream (*Diplodus sargus*). An observational study of the demography of juveniles of this species was carried out at six coves in Menorca Island (Balearic Islands, western Mediterranean). Three of the coves were located in the northern and wind exposed coast, at the Northeast (NE) side; while the other three were found along the southern and sheltered coast, at the Southwest (SW) side of the island. The settlement period extended from early May to late June and maximum juvenile densities at the sampling sites varied between 5 and 11 ind. m^-1^ with maximum values observed in late May simultaneously occurring in the two coasts. Our analysis of juvenile survival, based on the interpretation of the observed patters using an individual based model (IBM), revealed two stages in the size-mortality relationships. An initial density-dependent stage was observed for juveniles up to 20 mm TL, followed by a density independent stage when other factors dominated the survival at sizes > 20 mm TL. No significant environmental effects were observed for the small size class (<20mm TL). Different significant environmental effects affecting NE and SW coves were observed for the medium (20-30mm TL) and large (>30mm TL) size class. In the NE, the wind stress consistently affected the density of fish of 20–30 mm and >30 mm TL with a dome-shape effect with higher densities at intermediate values of wind stress and negative effect at the extremes. The best models applied in the SW coves showed a significant non-linear negative effect on fish density that was also consistent for both groups 20–30 mm and >30 mm TL. Higher densities were observed at low values of wave height in the two groups. Because of these variations, the number of juveniles present at the end of the period was unrelated to their initial density and average survival varied among locations. In consequence, recruitment was (1) primarily limited by denso-dependient procedures at settlement stage, and (2) by sea conditions at post-settlement, where extreme wave conditions depleted juveniles. Accordingly, regional hydrodynamic conditions during the settlement season produced significant impacts on the juvenile densities depending on their size and with contrasted effects in respectto cove orientation. The similar strength in larval supply between coves, in addition to the similar mean phenology for settlers in the north and south of the Island, suggests that all fish may come from the same parental reproductive pool. These factors should be taken into account when assessing relationships between settlers, recruits and adults of white seabream.

## Introduction

Dispersal, the process by which living organisms expand the space or range they live in, is one of the fundamental ecological processes affecting the dynamics of spatially structured populations [1]. In the marine realm, many benthic and necto-benthic organisms, belonging to multiple phyla, are characterized by an initial dispersal planktonic phase (i.e. eggs and larvae) followed by a more sedentary and site-attached benthic phase [2,3]. For most coastal marine species, early life-time dispersal is restricted to a relatively short pelagic phase (i.e. days to weeks) and, therefore, considerable research has focused on the processes shaping population connectivity at this stage [4,5]. Less it is known about the sedentary phase, in which larvae settle and early juveniles develop into adults. For littoral fish this stage is critically dependent on two early life history processes: i) settlement, i.e. the transition from the pelagic to the benthic environment, and ii) recruitment, where early juveniles join the adult population after development in nearshore areas [6,7]. Identifying the factors that influence survival during settlement and recruitment is essential in order to understand the replenishment and persistence of adult populations [4,8,9].

Recruitment to coastal fish populations is the final result of previous concatenated events which fish larvae have to overcome. Passive transport by currents, active swimming to settlement habitats and several post-settlement factors (both physical and biological) determine survival until recruitment [5,10–13]. The availability of suitable habitats (i.e. nursery areas) in which settlers can protect and grow becomes essential during the settlement and post-settlement phases [14]. However, other multiple co-occurring factors may influence survival throughout these stages. For example, it has been demonstrated that distance between spawning location and settlement sites is relevant for settler survival [15]. Also, survival in the post-settlement phase may be influenced by behavior, suitable microhabitats and prey availability, migration, and mortality related to the phenotypic-environmental mismatch [5,11,16–18]. During these two phases, some settlers and post-settlers may be particularly sensitive to unfavorable environmental conditions which can increase mortality rates [19–21].

The white seabream *Diplodus sargus* (distributed in the Eastern Atlantic, Mediterranean and Black Seas) is an ecologically and economically important species and is one of the most abundant fishes in sublittoral rocky reefs in the Mediterranean [22]. The information about different life history stages of this species is extensive, covering various aspects such as reproduction, growth, feeding, movement, larval and post-larval dispersal, habitat use and fisheries [23–30]. Moreover, a non-random, but size-selective mortality, has also been described for this species with indications to higher survival of larger individuals and an overall decrease in size variability over time at post-settlement stage [31,32]. However, the relative importance of environmental factors and biotic interactions, which may potentially modify the initial settlement and post-settlement patterns in determining recruits and adult population sizes, is still poorly understood.

In the Atlantic, white sea breams spawn from March to June at temperatures between 15 and 17°C [33] and release pelagic eggs that hatch after 3 days [34]. In the Mediterranean, spawning occurs slightly later, mostly in early May [31,32]. Larvae spend approximately between 13 and 28 days in the plankton, close to the sea surface [3,35,36] and post-larvae settle onto coastal habitats from mid-May to July, when they are 1 cm long [33,37,38]. Nursery habitats for these species are most commonly very shallow (<2 m depth) coves with gentle slope and heterogeneous substrata of sand, pebbles and rocks [39,40]. Previous research has demonstrated that the arrival of settlers to nursery coves is highly dependent on wind direction [38] and cove exposure [41] but post-settlement growth and survival have been suggested to be density-dependent processes [41,42]. While nearshore water temperature was shown to affect post-settlement growth and survival [38], the role of environmental drivers in these processes remains largely unknown. This includes direct effects of unfavorable environmental factors on settlers’ survival in the new habitats after settlement, an environmentally-induced displacement of settlers to unfavorable habitats uncoupled with the life history of fish (i.e. phenotypic-environmental mismatch [43]), or the synergies between these processes with an increased accessibility to predators [44].

The aim of this study is to investigate the factors (i.e. density-dependence, wind stress, wave height and coastal current velocity) driving the settlement patterns of the white seabream and determining its recruitment success in Menorca Island, Western Mediterranean. We hypothesize that a contrasting combination of environmental (i.e. wind stress, wave height and coastal current velocity) and biotic factors (i.e. fish density and size) could be determinant for the differences in the recruitment success between the north and south of the island. Knowledge on the ecological processes driving fish population dynamics during their early life stages and how they recruit into adult populations is essential for effective spatial management and conservation of these coastal fish species.

## Material and methods

### Study area

**Fig 1:**
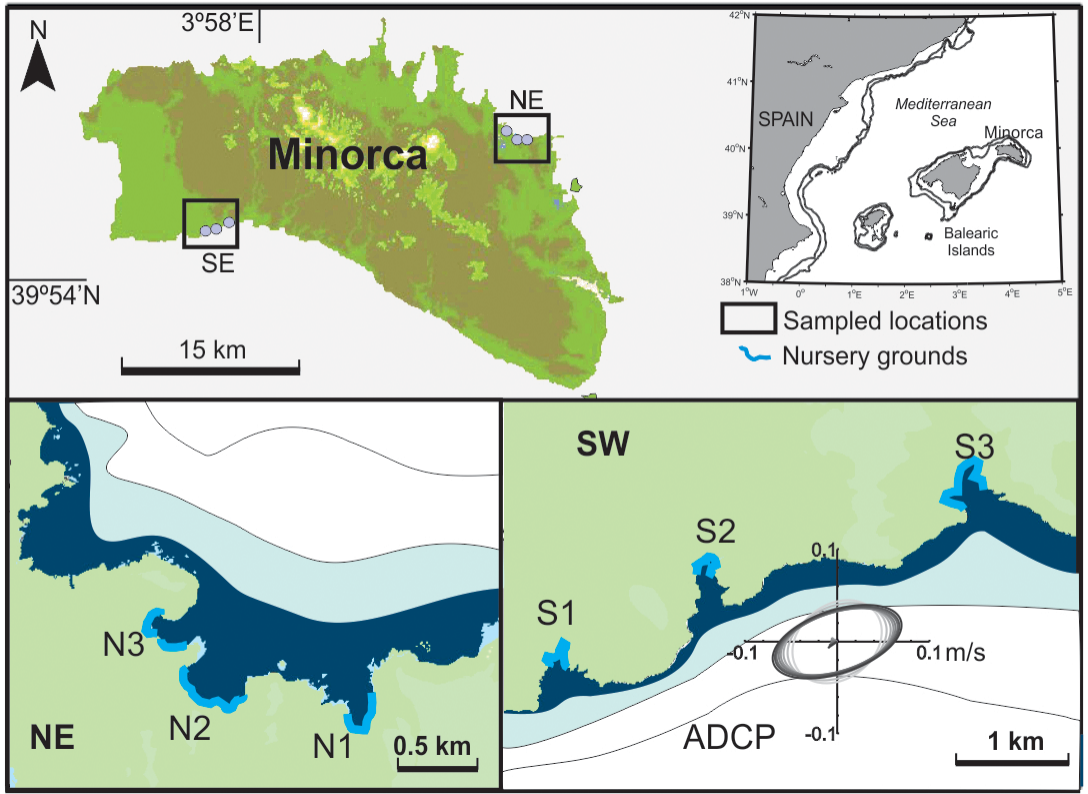
Study sites. The two distinct sampling locations at the Northeast (NE) and Southwest (SW) of the Menorca Island and the six coves monitored during the study. The Acoustic Doppler Current Profiler (ADCP) localization and the current variation ellipses measured at different depths (darker is shallower) are also indicated at the SW site. Maps were created by the authors by merging topographical maps generated with ArgGIS using IDEIB terrain elevation model (http://ideib.caib.es/visualitzador/visor.jsp) and the MATLAB software. There are no copyright restrictions associated and permissions for publication are not necessary.

The spatiotemporal dynamics of settlement were assessed at two locations in Menorca Island (Balearic Islands). The study period spanned from the 23^th^ of April to the 20^th^ of July 2012, in order to encompass the entire settlement period of the white seabream. As in the rest of the Balearic Archipelago, coastal circulation in the Balearics is mainly regulated by wind forcing, and the tidal component only represents a small fraction of the coastal flow [45]. The West-East elongated orientation of the island and the dominant northerly winds create two well-defined hydro-dynamical areas: the northern coast, exposed to the strongest storm episodes, and the more sheltered southern coast. Thus, the study focused on two locations, placed on the northeast and the southwest coast of the island (here named respectively NE and SW locations), and was performed at six coves (three per location) characterized by the presence of suitable microhabitats for white seabream settlement (Fig 1, Table 1). The sediment type of each cove showed in Table 1 was obtained from Pujol et al. 2013 [46]. The mouth of the coves is relatively similar (150–190 m), although two of the beaches N2 and S3 are more opened to the sea. Also, the coves in the southern coast (S1 to S3) are generally more elongated, offering a greater protection from rough seas. Since the sampling was not carried out in protected areas, nor did it involve studying of any endangered or protected species, prior research permits were not required.

**Table 1:** Location and geomorphological characteristics of the sampled coves and sampling characteristics. S: Sediment (CS Coarse Sand, MS Medium Sand); W: Width (m); L: Length (m); SL: Shoreline Length (m); A: Area (m^2^); NSD: Number of sampling days; ET: number of effective transects; TN: total number of white seabream juveniles censed during the study period; MNJ: maximum juveniles observed per day; TL: size ranges considering the Total Length (mm).

Code	Cove	S	W	L	SL	A	NSD	ET	TN	MNJ	TL
**N1**	Calderer	CS	180	118	453	14217	16	112	22432	5589	10–85
**N2**	S´Enclusa	MS	285	130	606	30199	17	184	41986	6148	10–65
**N3**	Mongofre	CS	158	184	623	29603	17	184	28833	4926	10–65
**S1**	Talaier	MS	204	141	409	11145	15	132	21281	5150	10–85
**S2**	Turqueta	MS	193	360	325	55493	15	89	21271	2844	10–85
**S3**	Macarella	MS	261	373	540	76300	15	156	36386	3347	10–90

### Environmental data

Wave and wind data were provided by the National Port Authority *Puertos del Estado*. Data from two meteorological buoys were used; one placed to the northeast (SIMAR-44-2083040: 40.00° N, 4.38° E) and, the other, located to the southwest of the study area (SIMAR-44-2079039: 39.88° N, 3.88° E). Additionally, at each cove, sea water temperatures were recorded during the entire study using an Onset HOBO Water Temp Pro v2 sensor moored on the seafloor at 5 m depth. Coastal currents were monitored with a bottom-mounted 1000 kHz Nortek Aquadopp Acoustic Doppler Current Profiler (ADCP) deployed at a depth of 27 m in the SW location. The sampling interval was set to 15 min. Currents were rotated so that *u* was the alongshore component.

### Underwater visual census

Abundance and size of white seabream juveniles (i.e. settlers and post-settlers) were assessed by means of Underwater Visual Census (UVC; [47]). At each cove, the population was sampled at midday (between 10 am and 4 pm) once or twice a week, depending on sea conditions. The three coves within each location were always monitored during the same sampling day. Juveniles were counted and size estimated along pre-defined 55 m long transects running parallel to the shoreline and covering the entire cove (the number of fixed transect varied between 6 and 11 depending on the shoreline length). The beginning and end of each transect were referred to some topographical feature of the coastline, to ensure accurate repeatability over time regardless of the observer. All censuses were carried out by the same three observers, who snorkeled slowly along transects extending from 0.5 to 3 m depth. Inter-comparisons between observers were carried out to reduce counting errors and disparities among them. The size of the individuals observed ranged from 10 mm to 100 mm total length (TL). The error of this size estimating method is ± 3.5 mm for *Diplodus* species [48]. The total number of juveniles censed per cove varied between 21271 and 41985 individuals (Table 1). For statistical analyses fish densities were standardised to juveniles per meter of shoreline.

### Juvenile population descriptors

White seabream juvenile population descriptors calculated for each of the six coves sampled were the total mean density (MD) and partial mean densities of individuals at total length (TL) of 10–20 mm (D_10-20_), 20–30 mm (D_20-30_) and ≥30 mm (D_>30_). These three size-class density intervals were established to better understand the evolution of the juvenile population size structure throughout the study period. Population peak (DP) was expressed as the maximum number of juveniles counted in one day throughout the whole sampling period and the recruitment level (RL) was referred to as the number of juveniles remaining at each cove at the end of the sampling period. Overall settler growth rate was calculated fitting the temporal variation of the population peak (see below). As an additional test to confirm whether the differences among coves and over time were significant, juvenile fish densities (MD, D_10-20_, D_20-30_, D_>30_) were analysed by means of Analysis of Variance with Repeated Measures ANOVAR [49]. This was the appropriate statistical test for the juvenile fish density data since the study involved repeated visits and monitoring the same pre-defined transects at each cove over time. Density values were Log transformed and the assumption of sphericity was tested and, when necessary, analysis under relaxed assumptions (i.e. using Greenhouse–Geisser, Huynh–Feldt and Lower-bound adjustments for ANOVAR) were applied. For conservative purposes statistical significant differences when considered when the probability (P) was higher than 0.001.

### Juvenile temporal population dynamics and environmental influence

Due to the large stochasticity in population of juveniles induced by natural and counting biases, we fitted a double sigmoid function to the temporal variation of density *l(t)*. This adjusted temporal settler variation was used as an input to the population model (see below). The equation used was:

$$l(t)=\frac{lmax}{2}\left[tanh(\frac{t+c1}{w})tanh(\frac{t-c2}{w})\right]$$

Where *lmax* is the maximum juvenile density, *c1* and *c2* are the slope parameters and *w* is the peak width.

To analyse the white seabream population dynamics, a juvenile population matrix, containing the number of individuals (*N*) at each size interval (*S*) over discrete time steps (*T*), was constructed by linearly interpolating the measured larval density variation over time in a regular time lapse (5 days) *vs* size range (5 mm) grid. Growth estimates (mm day^-1^) at each cove were calculated by robust regression fit of the temporal variation of the population at the larval peak. Mortality (M) rates were calculated by constructing survival curves using log transformed abundance-at-age data for each set of larvae settled within 5 day intervals.

$$M=(1-(N_{t}/N_{t-1})*100$$

Where *N*_*t*_ is the abundance of larvae at time *t* and *N*_*t-1*_ is the abundance of larvae at the previous time step. Despite the extensive larval (i.e. propagules) dispersal highlighted for this species [29] the method used assumes that emigration and immigration for the size range considered is negligible. Because of the particular geomorphology of the coves, it was reasonable to assume that settles and post-settles would have not emigrated from very shallow settlement habitats towards the deeper areas outside the coves. While this emigration from settlement habitat to deeper open coastal waters was not expected to be significant, late settler arrival (i.e. settlers of large size) could have also happened. Although this fact could have been a potential limitation of the method used, the incorporation of these newcomers to the population dynamics resulted in underestimation of instant mortality rates. Also, settlers with different larval histories could have suffered differential mortality as reported by Hamilton et al. (2008) [50].

An individual-based model was used for modeling settling and recruitment in each cove. The model tracked changes in the population at individual level over 170 days for each of the coves. Simulations, assumed a constant growth rate of the juveniles which was obtained as mentioned above. A random standard deviation of ± 0.05 mm day^-1^ was prescribed to the growth of each individual. According to previously mentioned mortality estimates, instant mortality rates for juveniles up to 20 mm were allowed to vary as a function of density and were adjusted to the mean value in the cove for larger sizes (see results). The model assumed that settlers started out all the same size, 10±2.5mm. Individuals longer than 55 mm were treated as recruits considering that they emigrate and do not die.

Given that both seasonal evolution (i.e. phenology) and the environmental drivers affecting settling dynamics were expected to be non-linear, we applied General Additive Modeling (GAM) to investigate their effects. GAMs are nonparametric regressions with the main advantage of not requiring a prior specification of underlying non-linear functional forms between dependent and independent variables [51]. Thus, the model allowed for the data to provide information about the shape of the functional relationships (smooth effects). To further analyse the non-linear dynamics of the settlement process and to strengthen the robustness of the analyses, density information of coves in the NE and SW of the island were respectively pooled and analysed separately in terms of the mean phenology and the main environmental drivers.

The GAM formulation applied was as following:

$$Log{\left(D\right)}_{t}=a+s(t)+\overset{j-m}{\underset{j}{\sum }}g_{j}(E_{t})+\varepsilon_{t}$$

where *D* is the density of fish at a given size range, *a* is the intercept, *E* is a vector of *m* environmental covariates (wind stress, wave height and coastal current velocity) at day *t*, *s* is a one-dimensional non-parametric smoothing function to capture the average phenology (no limit applied in the degrees of freedom), and *g* are one-dimensional non-parametric smoothing functions (cubic splines with up to a maximum of three degrees of freedom to avoid overfitting; i.e. four knots). ε denotes a Gaussian error term. We adopted a step-wise procedure to get a first initial GAM model removing one non-significant covariate at a time. To obtain the final model, model selection was based on the minimization Akaike’s information criterion (AIC). The best final model including environmental covariates is presented compared to the models including only the mean phenology. After the models were fitted, residuals were checked for homogeneity of variance, absence of temporal autocorrelation and violation of normality assumptions.

## Results

### Environmental conditions

**Fig 2:**
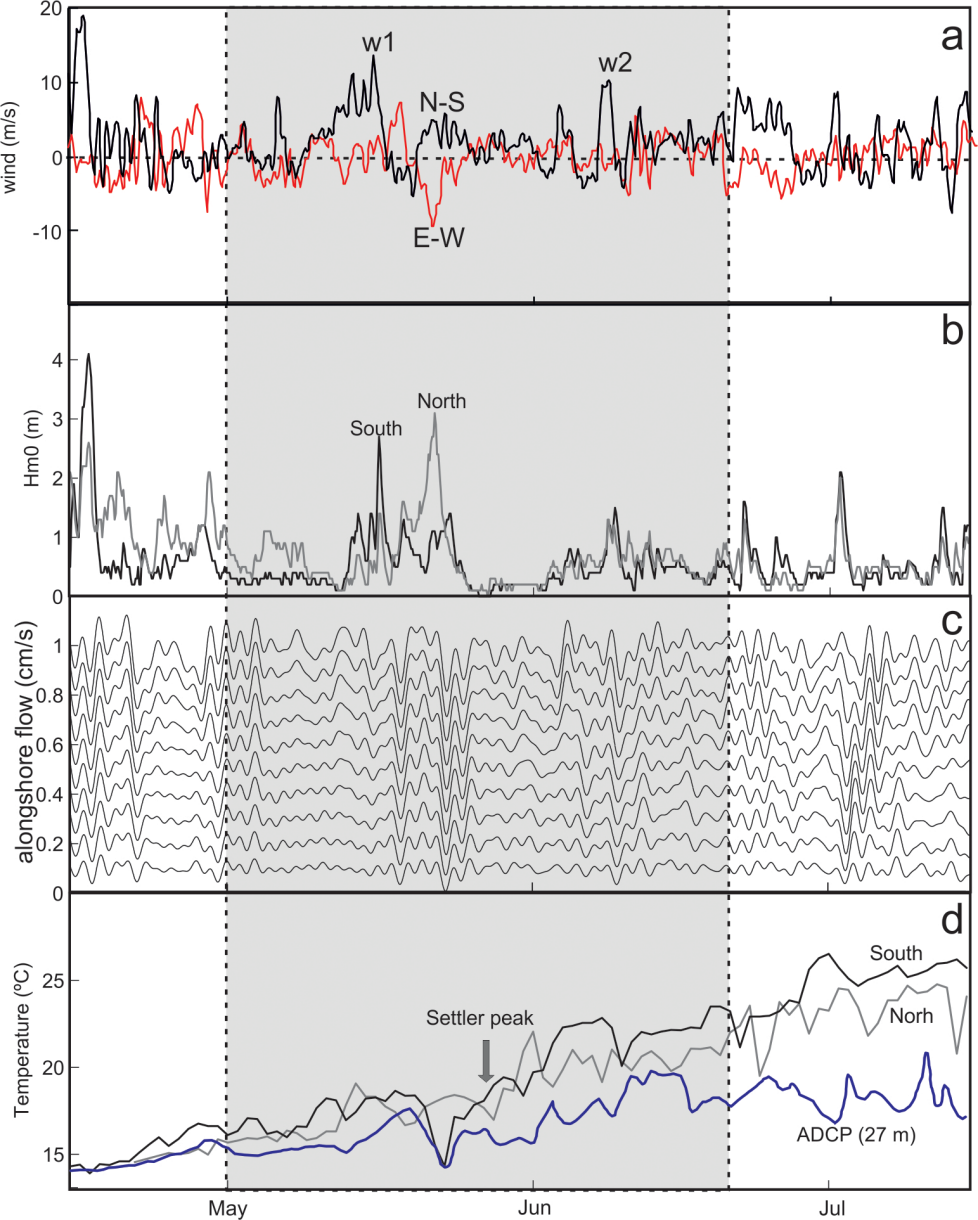
Environmental conditions. The shaded area indicates the sampling period encompassing the settlement and post-settlement period of the white seabream. a) Meridional (N-S back) and zonal (E-W red) components of the wind; b) wave height; c) alongshore oscillatory coastal currents; d) Seawater temperatures. The subsurface temperature (27 m) corresponds to that registered by de ADCP. W1 and W2 indicate two wind events with velocities above 10 ms^-1^.

Fig 2 summarises the environmental conditions in the coastal waters of Menorca during the survey period. The meridional component of the wind revealed the dominance of northerly winds (Fig 2A). Two stormy events, with wind intensities exceeding 10 ms^-1^ occurred during the study period. The first storm took place in May (w1), during the larvae arrival to the coves. This episode first increased the wave height from the south and, in the following days, increased wave height at the north (Fig 2B). A shorter storm event occurred in June (w2), when most settlers had already arrived. Wind pulses also carried the intensification of alongshore oscillatory coastal currents from typical values of 0–0.1 ms^-1^ to >0.15 ms^-1^ (Fig 2C). The shoreward component was significantly lower, displaying mean vector values of 0.03±0.02 ms^-1^, and negligible net component (<0.01 ms^-1^). Seawater temperature ranged from 14°C in April to more than 24°C in late July, with slightly higher temperatures at the south during summer (Fig 2D).

### Population descriptors

Mean population descriptors are shown in Table 2. Mean density (MD) ranged from 2.54±0.53 ind.m^-1^ to 5.94±0.97 ind.m^-1^ and density peak (DP) varied from 6.20 ind.m^-1^ to 12.38 ind.m^-1^. Around 80–90% of individuals of the maximum DP ranged between 10–15 mm TL. Results of repeated measures ANOVAR (S1 Table) showed that the data set did not satisfy the assumption of compound symmetry (Mauchly’s sphericity criterion); thus, there was need to proceed with the analysis under relaxed assumptions (i.e. using Greenhouse–Geisser, Huynh–Feldt and Lower-Bound corrected ANOVAR). No significant differences were obtained for MD between NE and SW coves, nor between-coves of the same location of the island. The difference in density between the NE and SW locations of the island was explained only for the significant differences obtained for juveniles larger than 30 mm TL with higher values in the SW than in the NE. No significant differences were obtained for the densities of smaller size classes (i.e. <20mm and 20-30mm TL). Mean growth obtained from the size-time evolution of the population also revealed statistically significant higher values in the SW coves than in the NE ones (F_1,4_ = 171.07; p<0.01) (Table 2).

**Table 2:** White seabream juvenile population descriptors (±S.D.) for each of the six coves sampled. MD: mean density; DP: density population peak; D_(10–20)_, D_(20–30)_ and D_(>30)_ are the mean densities of individuals of TL 10–20, 20–30, and ≥30 mm, respectively. Growth was calculated from the linear fitting of the temporal variation of the population peak. Densities expressed as ind.m^-1^ and growth expressed as mm.day^-1^.

Code	MD	DP	D_(10–20)_	D_(20–30)_	D_(>30)_	Growth
**N1**	3.11 ± 0.92	12.38	2.28 ± 0.84	0.69 ± 0.19	0.14 ± 0.04	0.43±0.03
**N2**	3.70 ± 0.80	10.15	3.19 ± 0.82	0.75 ± 0.18	0.14 ± 0.05	0.41±0.06
**N3**	2.73 ± 0.61	7.91	2.09 ± 0.61	0.52 ± 0.14	0.11 ± 0.03	0.43±0.07
**S1**	5.94 ± 0.97	12.60	3.99 ± 1.04	1.36 ± 0.45	0.59 ± 0.21	0.51±0.08
**S2**	4.36 ± 0.76	8.75	3.34 ± 0.89	0.65 ± 0.20	0.37 ± 0.14	0.48±0.10
**S3**	2.54 ± 0.53	6.20	1.48 ± 0.49	0.71 ± 0.24	0.43 ± 0.16	0.49±0.08

### Population dynamics, environmental drivers and survival rates

**Fig 3:**
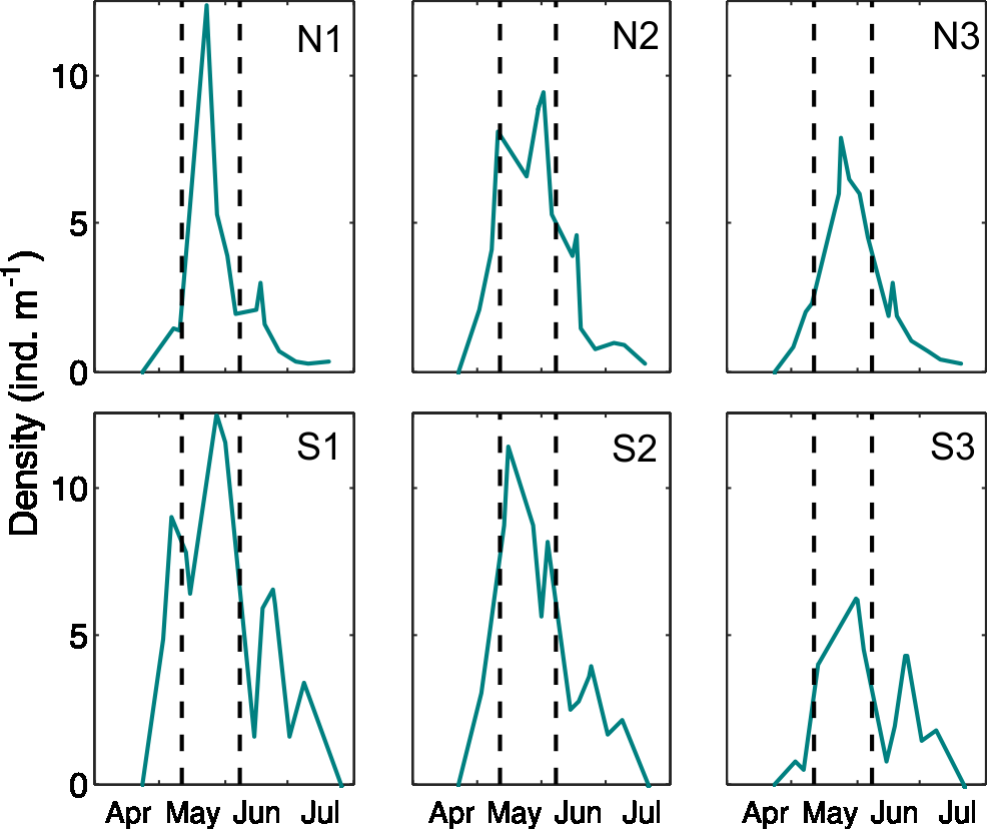
White seabream juvenile population density evolution. Temporal evolution of total juvenile densities at each cove. The dashed lines indicate the dates of storms w1 and w2 shown in Fig 2.

New white seabream settlers arrived at the coves in late April, when seawater temperatures at the settling sites where ~16°C. Population rapidly increased peaking in early or mid-May and decreasing therefrom until individuals were scarce in mid-July (<2 ind m^-1^) (Fig 3). With some variations, the patterns were similar in all six coves. Slight decreases in the population were observed in some cases (N2 and S1) after storm w1 but more intense population effects seemed to have occured after w2 when the population was composed by larger individuals (Fig 3).

**Fig 4:**
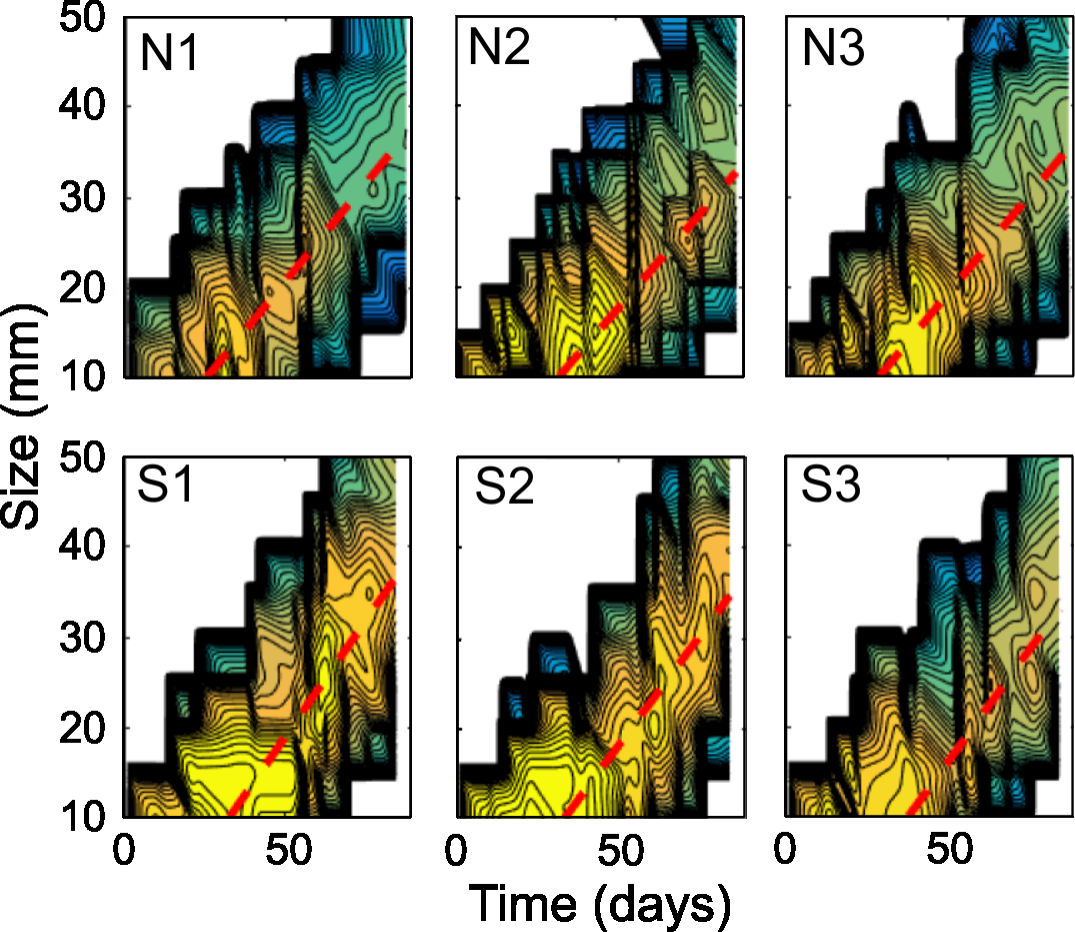
White seabream juvenile size-density evolution. Temporal size-density evolution of juveniles for each sampled cove. Contour colours are the logarithm of the estimated total population Log_10_ (ind). Dashed red lines indicate population growth rates (mm day^-1^).

The size-density temporal variation on the individuals presented different patterns depending on the cove, independently of their orientation (Fig 4). In S2 and S1 the maximum densities for seabreams of less than 20 mm TL were observed for almost 50 days from the onset of settlement. S1 also showed a second density peak of individuals of a size range between 25–30 mm TL at 65 days. By contrast, in N1 and N3 the maximum densities for sizes smaller than 20 mm TL were concentrated around 30 days from the onset of settlement. These two coves presented very low densities of larger sizes at the end of the sampling period. Higher densities for large sizes were observed only in S2 and S3. In N1 and S3 the density for all the sizes smaller than 25 mm TL was lower than in the rest of the coves. Finally, fish of larger sizes (>30 mm TL) presented lower densities from the day 70 in the coves N1 and N3.

Around 80–90% of individuals of the maximum population peak ranged between 10–20 mm TL. The adjusted function revealed the general trend at each cove (S1 Fig, S2 Table). Two general patterns detached from the cove orientation (i.e. north-south orientation) were observed. Coves N2, S1 and S3 presented fast accumulation rates but truncated shapes, whereas a slower accumulation was observed at N1, N3 and S3. The settlement rate revealed different settling peaks throughout the 60 day period and the six coves presented different patterns, which were not related to their north-south orientation (S1 Fig). For example, 3 settling peaks were observed at S1, S2 and N2 whereas N1, N3 and S3 presented a single peak. The peaks at 20 and 42 days are consistent with the coastal current intensification episodes occurring at the beginning and mid-May (see Fig 2).

**Fig 5:**
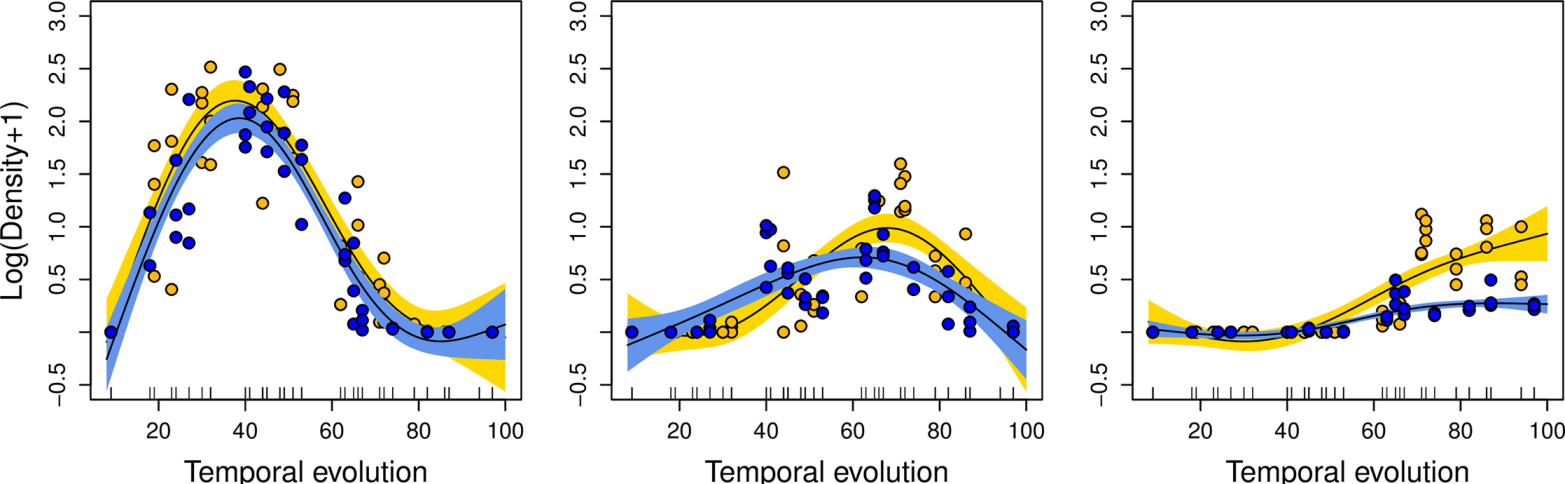
General additive model for white seabream density segregated by size interval. Mean phenological pattern for each fish size group (10–20 mm, left; 20–30 mm, center; >30 mm, right) and cove orientation (North, blue; South, yellow). Fitted lines (solid line), 95% confidence intervals (grey shaded areas) and residuals (dots) are shown. Note that each line represents a different model (Table 4), which are overlapped for each fish size group.

When fish of all sizes were analysed together, general additive modelling captured the dome-shaped functional form of the settling dynamics with a similar mean maximum of density around day 41 for both sides of the island but with a faster decrease in density in NE than in the SW coves (S2 Fig). However, the separation of information per size class revealed contrasting phenological patterns as well as environmental drivers between coves in the NE and the SW. Model results showed that the global phenology (S2 Fig) mainly reflected the dynamics observed for small fish (TL < 20 mm) with a maximum density at day 40 and the same rate of density decrease for both sides of the island (Fig 5). For fish 20–30 mm TL, the temporal evolution showed a delayed maximum (at 70 days) and a higher density in the SW of the island. This pattern contrasted with the dynamics observed in the NE that displayed a maximum at 60 days and higher density earlier in the settling process (ca. at 40 days). For fish >30 mm TL, the higher densities were observed at the end of the settling process (ca. 100 days) with considerable higher values in the SW compared with the NE (Fig 5). Accordingly, the recruitment level (i.e. the number of juveniles at the end of the sampling period) was higher in the SW than in the NE (Table 3).

**Table 3:** Population dynamics parameters. Recruitment level (RL) of white seabream juveniles at the end of the sampling period indicating the mortality rate (M) and the proportion of juveniles that survive until recruitment size (% S) resulting from model adjustment to the censed recruits at each cove.

Code	RL (ind. m^-1^)	M (ind. day^-1^)	% S
**N1**	0.28	0.038	2.58
**N2**	0.39	0.039	1.26
**N3**	0.28	0.037	1.90
**S1**	1.57	0.032	4.93
**S2**	0.69	0.040	2.47
**S3**	0.54	0.036	3.92

**Fig 6:**
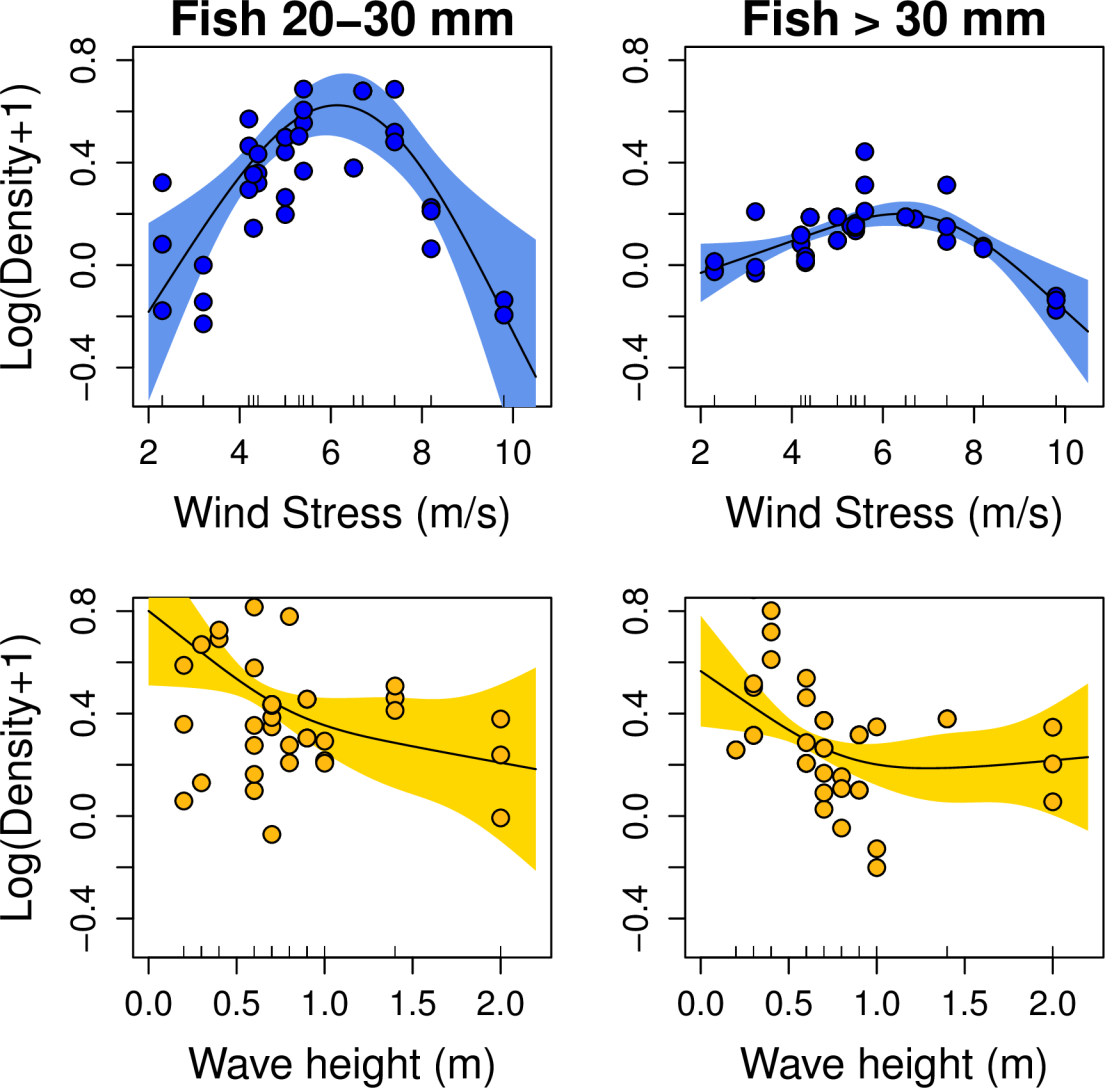
Best general additive model for white seabream density. Main environmental drivers for 20–30 mm (left column) and >30 mm (right column) group size obtained from the best General Additive Model (Table 4) including the phenology effect. Plots above (blue) represent the best models for the northeast coves, and plots below (yellow) the best for the southwest coves. Fitted lines (solid line), 95% confidence intervals (colour shaded areas) and residuals (dots) are shown.

No significant environmental effects were observed for the small size class (<20mm TL) (Table 4), consistent with the models fitted with all data pooled (S2 Fig). However, different significant environmental effects affecting NE and SW coves were observed for the medium (20-30mm TL) and large (>30mm TL) size classes. In the NE, the wind stress consistently affected the density of fish of 20–30 mm and >30 cm TL with a dome-shape effect and, thus, higher densities at intermediate values of wind stress (ca. 4–8 m/s) and negative effect in the extremes (Fig 6, Table 4). By contrast, the best models applied in the SW coves showed a significant non-linear negative effect on fish density which was also consistent for both groups 20–30 mm and >30 mm TL. Higher densities were observed at low values of wave height in the two groups (Fig 6, Table 4).

**Table 4:** Best general additive models (GAMs). GAMs obtained for fish larvae density (D) of Northeast and Southwest coves, and for each group of fish size (10–20 mm, 20–30 mm and >30 mm). Best model was chosen based on the minimization of the Alkaike Information Criterion (AIC) score. *a*: intercept, *s*: 1- dimensional smoothing functions, *t*: day; *WS*: Wind Stress; *H*: Wave Height; *ε* error term. DE (%): percentage of deviance explained.

Model structure	DE (%)	AIC
**North**		
Fish 10–20 mm		
*Log*(*D*)_*t*_ = *a* + *s*(*t*) + *ε*_*t*_	87.8	28.68
Fish 20–30 mm		
*Log*(*D*)_*t*_ = *a* + *s*(*t*) + *ε*_*t*_	54.7	14.74
*Log*(*D*)_*t*_ = *a* + *s*(*t*) + *s*(*WS*_*t*_) + *ε*_*t*_	63.6	8.09
Fish > 30 mm		
*Log*(*D*)_*t*_ = *a* + *s*(*t*) + *ε*_*t*_	69.4	-94.10
*Log*(*D*)_*t*_ = *a* + *s*(*t*) + *s*(*WS*_*t*_) + *ε*_*t*_	77.1	-102.75
**South**		
Fish 10–20 mm		
*Log*(*D*)_*t*_ = *a* + *s*(*t*) + *ε*_*t*_	81	54.29
Fish 20–30 mm		
*Log*(*D*)_*t*_ = *a* + *s*(*t*) + *ε*_*t*_	62.4.7	31.88
*Log*(*D*)_*t*_ = *a* + *s*(*t*) + *s*(*H*_*t*_) + *ε*_*t*_	68.5	27.17
Fish > 30 mm		
*Log*(*D*)_*t*_ = *a* + *s*(*t*) + *ε*_*t*_	71.08	-4.04
*Log*(*D*)_*t*_ = *a* + *s*(*t*) + *s*(*H*_*t*_) + *ε*_*t*_	76.7	-8.73

The life history of white seabream presented two diferent phases related to mortality. During the first period, when most of the settlers were between 10 and 20 mm TL, mortality was density-dependent (r^2^ = 0.59) (S3 Fig). This relationship was not maintained at larger size-classes (TL > 20 mm) as confirmed by the results of the non-linear regressions. Results from model adjustment to the observed recruits at each cove yielded mortality rates of these larger post-settlers of 0.033 to 0.040 ind. day^-1^ (Table 3). The proportion of juveniles which survived to recruitment size varied between 1.26 and 4.93%.

## Discussion

The settlement dynamics of the juvenile white seabream at the coves situated in the northeast and southwest coast of Menorca indicates that processes driving settlement and post-settlement operate at an island scale (i.e. settlers arriving at the coves in late April, population rapidly increasing until mid-May and decreasing until mid-July, ubiquitously). Nevertheless, some cove specificities have been found mainly for the size-density temporal evolution, with coves presenting different: i) new settlement (juveniles <20 mm TL) peak amplitudes, ii) phenologies for medium (juveniles between 20-30mm TL) and large juveniles (>30mm TL), and iii) post-settlement densities and survival rates. We demonstrate that for new settlers the timing of arrival to the coves is also critical for juvenile survival at the end of the recruitment period. Thus, most of the settlers arriving at the main peak were removed from the population mainly because mortality was a density-dependent process exponentially increasing with abundance but only for new settlers. By contrast, factors other than abundance seem to regulate mortality for larger juveniles in a non-linear relationship.

Our results revealed that regional hydrodynamic conditions during the settlement season produced a significant impact on the juvenile densities depending on their size and with contrasted effects in respect to cove orientation. Some differences where observed between coves at the same part of the island in terms of density accumulation rates and number of settling peaks but, as a whole, the strength of larval supply was similar within the northeast and the southwest of the island. Although spatial variability in fish settlement peak between sites of a given locality has already been reported elsewhere [52–54], this similar strength in larval supply, in addition to the similar mean phenology for settlers in the north and south of the Island, suggests that all fish may come from the same parental reproductive pool or spawning population, while larval dispersal from multiple spawning areas can also be considered [28].

A slight decrease in density was observed after the two storms registered during the settlement period. The first storm occurring in mid-May could have had a major effect on new settlers but no direct relationships were detected between these new settlers and environmental conditions. However, the environmental effect at this early stage of the settlement process is negligible compared to the influence of density dependence and the contrasting response of settlers’ size at each cove. The second storm occurred in early-June when most of the juveniles ranged 20–30 mm TL. For this size range, and also for larger ones, the environment impacted the population at a larger scale with consistent but different effects observed in each side of the island. The increase in wind stress and wave height clearly intensified the water turbulence near the cost and might have modified the behaviour of juveniles. After the second storm, density rapidly recovered in the south coves but not in the north ones, suggesting a contrasting environmental effect between the north and the south of the island. In the south, storms likely dispersed individuals over a broader area of the cove but did not cause significant mortality with higher wave height related to lower densities of medium and large size individuals. By contrast, the dominance of northerly winds during the settling period seemed to have had a major effect on medium and large juveniles growing in the northeast coves. In this case, high and low values of meridional wind stress were associated with extreme conditions which could have negatively affected the survival of settlers, and only intermediate values favoured the survival and the consequent recruitment. This is known in fisheries oceanography as the ‘Optimal Environmental Window’, which was an ecological hypothesis initially defined and commonly observed for pelagic species with survival or early life stages highly dependent on the wind patterns [55]. In essence, this is the oceanographic extension on the ‘Intermediate Disturbance Hypothesis’ [56], a classic pattern known in ecology stating that key ecological processes, species fitness or species diversity are favoured at intermediate values on natural or anthropogenic levels of disturbance. We suggest that strong meridional winds displace settlers to unfavourable habitats [43] in which, among other factors, predation effect can increase. Similarly we also observed a delay in the juveniles’ peak of medium and large juveniles of the coves situated in the northeast coast resulting in higher densities of large juveniles, and in consequence higher survival rates in the southwest cost of the island. This observation is consistent with directional size selective mortality observed in reef fish [57] potentially expected in an unfavourable environment [43]. All these spatial differences suggest the importance of site-specific conditions for settlement, in this case related with the orientation and exposition of each cove with respect to wave direction and hydrodynamics, habitat quality and biological interactions (e.g., predation, competition) [38,41,58–60].

Félix-Hackradt et al. (2013b)[61] showed a strong influence of local winds and currents on larval supply to nursery areas of species of the same genus. Vigliola (1998) [38], also argued that wind regimes regulate white seabream settlement in Marseilles Bay (France), and that coastward winds from the sea favoured settlement of this species, due to the superficial position of its larvae in the water column. By contrast, Raventos and Macpherson (2005) [62] found that calm weather favoured settlement for another necto-benthic species with pelagic larvae (*Symphodus* spp.) in the Spanish Catalan coast.

Habitat quality is mainly determined by habitat structure because it can cause different mortality and/or growth processes, which in turn result in a spatial variability of fish settlement peak [61,63–66], a theory with potential relevance for our study. For example, the coves situated in the southwest coast of the island presented higher survival rates than in the northeast;Es Talaier (S1) presented the highest settlement peak, as well as the most heterogeneous substratum among the coves in the study and this may have favoured more refuges and hence higher survival rates and settlement success.

Settlement peak densities shown in the present study ranged between 6 ind.m^-1^ and 13 ind.m^-1^, which are among the highest peak density values reported for white seabream in the Mediterranean Sea (Table 5). However, contrastingly, the recruitment levels were very low in all cases and were comparable to other Mediterranean areas (<2 ind.m^-1^) (see references in Table 5), which was a consequence of density-dependent mortality processes regulating the initial settlement at higher levels compared with other locations. This is in accordance with the density-dependent mortality reported for *Diplodus* spp. (including white seabrams) [65,66]. In our study, 87 to ~97% of the juveniles disappeared 50–60 days after the settlement peak. Such mortality values are similar to those reported in other Mediterranean regions for the same time period. Arceo et al. (2012) [67] found mortalities of up to 60 to ~ 99% in the Cap Roux Fishery Reserve and adjacent areas in Saint-Raphaël (France) in 2011. Similarly, Macpherson et al [65] found mortalities from 50 to ~99% in Gerona (Spain) and in Marseille (France) in 1994–1995.

**Table 5:** Different maximum settlement population peaks (PP) reported in several areas of the Mediterranean. Locations: 1) Marseille (France); 2) Girona (Spain); 3) Banyuls (France); 4) Portofino (Italy); 5) Elba (Italy); 6) French Catalan coast (France); 7) Apulian Adriatic coast (Italy); 8) Cap Roux Fishery Reserve and adjacent areas in Saint-Raphaël (France); 9) Menorca island (Spain).

Location	PP (ind.m^-1^)	Year	References
**1**	16	1993–1995	[3] 1997; Vigliola 1998
**2**	7	1993–1995	[3]
**3**	>2	1994–1995	[3]
**4**	4	1994–1995	[3]
**5**	>3	1994–1995	[3]
**6**	>4	2005–2007	[68]
**7**	4	2009–2010	[69]
**8**	>2	2011	[67]
**9**	12	2012	This study
**9**	>5	2013	[41]

The importance of post-settlement processes compared to other demographic processes determining adult local population densities is not clear [9,70] and only a few studies have simultaneously considered multiple life stages across different spatial scales. For instance, Di Franco et al., (2013) [69] found no significant relationships between the density of adults, settlers, recruits and young of the year of white seabream, and attributed it to a possible decoupling in space between the sequential life history stages of fish caused by dispersal processes through sea currents or active fish movements. However, other studies found a significant relationship between the density of settlers or late juveniles and the recruitment levels of *Diplodu*s spp. [66,71].In spite of the high settlement values we reported here, the densities of adults of white seabream in Menorca Island were very low compared with those in other Mediterranean regions [22,72–74]. Cardona et al. (2007) [75] hypothesized that white seabream density in Menorca was low because of the oligotrophy of the coastal waters around the island, which makes the population ecologically adapted to be controlled by low density-dependent mortality as evidenced by the present study. Thus, the results reported here indicate that post-settlement processes at nursery habitats are more likely the limiting factor. Nevertheless, further studies are necessary to assess the temporal and spatial relationships between juvenile and adult white seabream.

In conclusion, synchronous dynamics were reported at the scale of Menorca Island for the settlement of white seabreams, highlighting the importance of island-scale processes regulating this essential ecological process for fish population dynamics. Although the intensity of settlement peaks was variable among coves, juvenile density at the end of settlement period (i.e. recruitment level) was globally low at all coves, suggesting that density-dependent mortality levelled initial differences in settlement. The contrasting phenology observed for medium and large individuals revealed different effects of environmental drivers affecting the survival rates at the end of the settlement period between coves situated at both locations of the island. Consequently, these results highlight that matching of larval release and dispersal with specific climatic conditions may strongly underpin and shape the success of settlement (match-mismatch hypothesis [76]). Future research on the influence of environmental variables on fish life history should assess the related spatial and temporal variability at multiple scales and at multiple life phases in order to better account for their possible influences on final adult population replenishment.

## Supporting information

S1 Table Results of repeated measures ANOVA comparing juvenile total density and partial densities of individuals at total length (TL) of 10–20 mm (D_10-20_), 20–30 mm (D_20-30_) and ≥30 mm (D_>30)_ over time (Sampling days, SD) at different orientations (Northeast and Southwest) and the coves (three in each orientation) off Menorca Island. SS: Sum of Squares; DF: Degrees of Freedom; MS: Mean Squares; F: Statistic; P: Probability; W: Statistic for the Mauchly's Test of Sphericity; GG: Greenhouse-Geisser correction; HF: Huynh-Feldt correction; LB: Lower-Bound estimate; E: Epsilon; ADF: Adjusted Degrees of Freedom (1–2); AP: Adjusted Probability. For conservative purposes statistical significant differences when considered when the probability (P) was higher than 0.001.
(DOCX)

S2 Table Parameters of the curve fitting settlers (10–20 mm) variations at each cove. *Lmax* is the maximum juvenile density, *C* is the slope parameters, *w* is the peak width and r^2^ the correlation coefficient.
(DOCX)

S1 Fig White seabream settler densities time-evolution (left) and rate of settler arrivals (right). Left panels: measured settler (10–20 mm) density variation (dots and squares) and adjusted double sigmoid functions (lines). Right panels: values are indicated as daily % of total settlers arriving to each cove.
(TIF)

S2 Fig General additive model of settling dynamics of the whole white seabream juvenile population. Blue represents the northeast coves, and yellow the southwest coves. Fitted lines (solid line), 95% confidence intervals (color shaded areas) and residuals (dots) are shown.
(TIF)

S3 Fig White seabream mortality rate. Density-dependent mortality relationship for juveniles smaller than 20 mm including all the individuals counted in the six coves. The vertical axis represents the daily mortality rate in percentage.
(TIF)
