# Supplementary material for: Settlement and post-settlement survival rates of the white seabream (Diplodus sargus) in the western Mediterranean Sea
Source: PLoS One. 2018 Jan 11;13(1):e0190278. doi: 10.1371/journal.pone.0190278 (PMC5764285; doi:10.1371/journal.pone.0190278)
Supplement: S1 Table — SS: Sum of Squares; DF: Degrees of Freedom; MS: Mean Squares; F: Statistic; P: Probability; W: Statistic for the Mauchly's Test of Sphericity; GG: Greenhouse-Geisser correction; HF: Huynh-Feldt correction; LB: Lower-Bound estimate; E: Epsilon; ADF: Adjusted Degrees of Freedom (1–2); AP: Adjusted Probability. For conservative purposes statistical significant differences when considered when the probability (P) was higher than 0.001. (DOCX) [file pone.0190278.s001.docx]

| **Juvenil Total Density** | | | | | | | | | | | | | | | |
| --- | --- | --- | --- | --- | --- | --- | --- | --- | --- | --- | --- | --- | --- | --- | --- |
|  | **SS** | **DF** | **MS** | **F** | **P** |  |  |  |  |  |  |  |  |  |  |
| **Intercept** | 193,960 | 1 | 193,960 | 208,357 | <0,001 |  |  |  |  |  |  |  |  |  |  |
| **Orientation (O)** | 0,010 | 1 | 0,010 | 0,010 | 0,919 |  |  |  |  |  |  |  |  |  |  |
| **Cove (C)** | 2,465 | 2 | 1,232 | 1,324 | 0,275 |  |  |  |  |  |  |  |  |  |  |
| **O*C** | 3,967 | 2 | 1,984 | 2,131 | 0,130 |  |  |  |  |  |  |  |  |  |  |
| **Error** | 45,614 | 49 | 0,931 |  |  |  |  |  |  |  |  |  |  |  |  |
| Mauchly’s sphericity criterion for Samplig Day (SD), W = 0.001, DF=90 (P < 0.001) | | | | | | | | | | | | | | | |
|  |  |  |  | **GG** | | | | **HF** | | | | **LB** | | | |
|  | **DF** | **F** | **P** | **E** | **ADF1** | **ADF2** | **AP** | **E** | **ADF1** | **ADF2** | **AP** | **E** | **ADF1** | **A DF2** | **AP** |
| **SD** | 13 | 17,947 | <0,001 | 0,508 | 6,599 | 323,353 | <0,001 | 0,655 | 8,513 | 417,124 | <0,001 | 0,077 | 1,000 | 49,000 | <0,001 |
| **SD*O** | 13 | 2,956 | <0,001 | 0,508 | 6,599 | 323,353 | 0,006 | 0,655 | 8,513 | 417,124 | 0,003 | 0,077 | 1,000 | 49,000 | 0,092 |
| **SD*C** | 26 | 2,177 | <0,001 | 0,508 | 13,198 | 323,353 | 0,010 | 0,655 | 17,025 | 417,124 | 0,004 | 0,077 | 2,000 | 49,000 | 0,124 |
| **SD*O*C** | 26 | 2,505 | <0,001 | 0,508 | 13,198 | 323,353 | 0,003 | 0,655 | 17,025 | 417,124 | <0,001 | 0,077 | 2,000 | 49,000 | 0,092 |
| **Error** | 637 |  |  |  |  |  |  |  |  |  |  |  |  |  |  |
| **Juvenil Partial Density 10-20** | | | | | | | | | | | | | | | |
|  | **SS** | **DF** | **MS** | **F** | **P** |  |  |  |  |  |  |  |  |  |  |
| **Intercept** | 115,045 | 1 | 115,045 | 150,477 | 0,000 |  |  |  |  |  |  |  |  |  |  |
| **Orientation (O)** | 0,384 | 1 | 0,384 | 0,503 | 0,482 |  |  |  |  |  |  |  |  |  |  |
| **Cove (C)** | 2,190 | 2 | 1,095 | 1,432 | 0,249 |  |  |  |  |  |  |  |  |  |  |
| **O*C** | 2,997 | 2 | 1,498 | 1,960 | 0,152 |  |  |  |  |  |  |  |  |  |  |
| **Error** | 37,462 | 49 | 0,765 |  |  |  |  |  |  |  |  |  |  |  |  |
| Mauchly’s sphericity criterion for Samplig day (SD), W = 0.027, DF=54 (P < 0.001) | | | | | | | | | | | | | | | |
|  |  |  |  | **GG** | | | | **HF** | | | | **LB** | | | |
|  | **DF** | **F** | **P** | **E** | **ADF1** | **ADF2** | **AP** | **E** | **ADF1** | **ADF2** | **AP** | **E** | **ADF1** | **A DF2** | **AP** |
| **SD** | 10 | 20,689 | 0,000 | 0,579 | 5,788 | 283,611 | 0,000 | 0,732 | 7,321 | 358,711 | 0,000 | 0,100 | 1,000 | 49,000 | 0,000 |
| **SD*O** | 10 | 5,084 | 0,000 | 0,579 | 5,788 | 283,611 | 0,000 | 0,732 | 7,321 | 358,711 | 0,000 | 0,100 | 1,000 | 49,000 | 0,029 |
| **SD*C** | 20 | 2,471 | 0,000 | 0,579 | 11,576 | 283,611 | 0,005 | 0,732 | 14,641 | 358,711 | 0,002 | 0,100 | 2,000 | 49,000 | 0,095 |
| **SD*O*C** | 20 | 2,680 | 0,000 | 0,579 | 11,576 | 283,611 | 0,002 | 0,732 | 14,641 | 358,711 | 0,001 | 0,100 | 2,000 | 49,000 | 0,079 |
| **Error** | 490 |  |  |  |  |  |  |  |  |  |  |  |  |  |  |
| **Juvenil Partial Density 20-30** | | | | | | | | | | | | | | | |
|  | **SS** | **DF** | **MS** | **F** | **P** |  |  |  |  |  |  |  |  |  |  |
| **Intercept** | 30,722 | 1 | 30,722 | 205,026 | 0,000 |  |  |  |  |  |  |  |  |  |  |
| **Orientation (O)** | 0,067 | 1 | 0,067 | 0,448 | 0,507 |  |  |  |  |  |  |  |  |  |  |
| **Cove (C)** | 0,590 | 2 | 0,295 | 1,968 | 0,151 |  |  |  |  |  |  |  |  |  |  |
| **O*C** | 0,773 | 2 | 0,386 | 2,578 | 0,086 |  |  |  |  |  |  |  |  |  |  |
| **Error** | 7,342 | 49 | 0,150 |  |  |  |  |  |  |  |  |  |  |  |  |
| Mauchly’s sphericity criterion for Samplig day (SD), W = 0.035, DF=65 (P < 0.001) | | | | | | | | | | | | | | | |
|  |  |  |  | **GG** | | | | **HF** | | | | **LB** | | | |
|  | **DF** | **F** | **P** | **E** | **ADF1** | **ADF2** | **AP** | **E** | **ADF1** | **ADF2** | **AP** | **E** | **ADF1** | **A DF2** | **AP** |
| **SD** | 11 | 29,773 | 0,000 | 0,633 | 6,966 | 341,330 | 0,000 | 0,824 | 9,067 | 444,287 | 0,000 | 0,091 | 1,000 | 49,000 | 0,000 |
| **SD*O** | 11 | 6,348 | 0,000 | 0,633 | 6,966 | 341,330 | 0,000 | 0,824 | 9,067 | 444,287 | 0,000 | 0,091 | 1,000 | 49,000 | 0,015 |
| **SD*C** | 22 | 2,852 | 0,000 | 0,633 | 13,932 | 341,330 | 0,000 | 0,824 | 18,134 | 444,287 | 0,000 | 0,091 | 2,000 | 49,000 | 0,067 |
| **SD*O*C** | 22 | 3,463 | 0,000 | 0,633 | 13,932 | 341,330 | 0,000 | 0,824 | 18,134 | 444,287 | 0,000 | 0,091 | 2,000 | 49,000 | 0,039 |
| **Error** | 539 |  |  |  |  |  |  |  |  |  |  |  |  |  |  |
| **Juvenil Partial Density >30** | | | | | | | | | | | | | | | |
|  | **SS** | **DF** | **MS** | **F** | **P** |  |  |  |  |  |  |  |  |  |  |
| **Intercept** | 8,007 | 1 | 8,007 | 87,206 | 0,000 |  |  |  |  |  |  |  |  |  |  |
| **Orientation (O)** | 1,785 | 1 | 1,785 | 19,443 | 0,000 |  |  |  |  |  |  |  |  |  |  |
| **Cove (C)** | 0,262 | 2 | 0,131 | 1,426 | 0,250 |  |  |  |  |  |  |  |  |  |  |
| **O*C** | 0,182 | 2 | 0,091 | 0,993 | 0,378 |  |  |  |  |  |  |  |  |  |  |
| **Error** | 4,499 | 49 | 0,092 |  |  |  |  |  |  |  |  |  |  |  |  |
| Mauchly’s sphericity criterion for Samplig day (SD), W = 0.101, DF=20 (P < 0.001) | | | | | | | | | | | | | | | |
|  |  |  |  | **GG** | | | | **HF** | | | | **LB** | | | |
|  | **DF** | **F** | **P** | **E** | **ADF1** | **ADF2** | **AP** | **E** | **ADF1** | **ADF2** | **AP** | **E** | **ADF1** | **A DF2** | **AP** |
| **SD** | 6 | 25,101 | 0,000 | 0,656 | 3,936 | 192,851 | 0,000 | 0,793 | 4,759 | 233,195 | 0,000 | 0,167 | 1,000 | 49,000 | 0,000 |
| **SD*O** | 6 | 4,966 | 0,000 | 0,656 | 3,936 | 192,851 | 0,001 | 0,793 | 4,759 | 233,195 | 0,000 | 0,167 | 1,000 | 49,000 | 0,030 |
| **SD*C** | 12 | 0,852 | 0,597 | 0,656 | 7,871 | 192,851 | 0,557 | 0,793 | 9,518 | 233,195 | 0,574 | 0,167 | 2,000 | 49,000 | 0,433 |
| **SD*O*C** | 12 | 0,893 | 0,555 | 0,656 | 7,871 | 192,851 | 0,522 | 0,793 | 9,518 | 233,195 | 0,537 | 0,167 | 2,000 | 49,000 | 0,416 |
| **Error** | 294 |  |  |  |  |  |  |  |  |  |  |  |  |  |  |
