# Supplementary material for: Settlement and post-settlement survival rates of the white seabream (Diplodus sargus) in the western Mediterranean Sea
Source: PLoS One. 2018 Jan 11;13(1):e0190278. doi: 10.1371/journal.pone.0190278 (PMC5764285; doi:10.1371/journal.pone.0190278)
Supplement: S2 Table — Lmax is the maximum juvenile density, C is the slope parameters, w is the peak width and r2 the correlation coefficient. (DOCX) [file pone.0190278.s002.docx]

| **Cove code** | ***Lmax* (ind.)** | ***C*** | ***w*** | **r^2^** | **Max dens. (ind./m)** |
| --- | --- | --- | --- | --- | --- |
| **N1** | 4200±1950 | 5.4 | 8.5 | 0.87 | 9.3 |
| **N2** | 4968±469 | 19.1 | 4.8 | 0.96 | 8.2 |
| **N3** | 4146±800 | 13.1 | 8.2 | 0.98 | 6.7 |
| **S1** | 3506±515 | 20.2 | 1.7 | 0.90 | 8.6 |
| **S2** | 2618±351 | 20.5 | 5.5 | 0.94 | 8.0 |
| **S3** | 2277±285 | 16.1 | 2.9 | 0.95 | 4.2 |
