# Supplementary figures and images for: Settlement and post-settlement survival rates of the white seabream (Diplodus sargus) in the western Mediterranean Sea
Source: PLoS One. 2018 Jan 11;13(1):e0190278. doi: 10.1371/journal.pone.0190278 (PMC5764285; doi:10.1371/journal.pone.0190278)

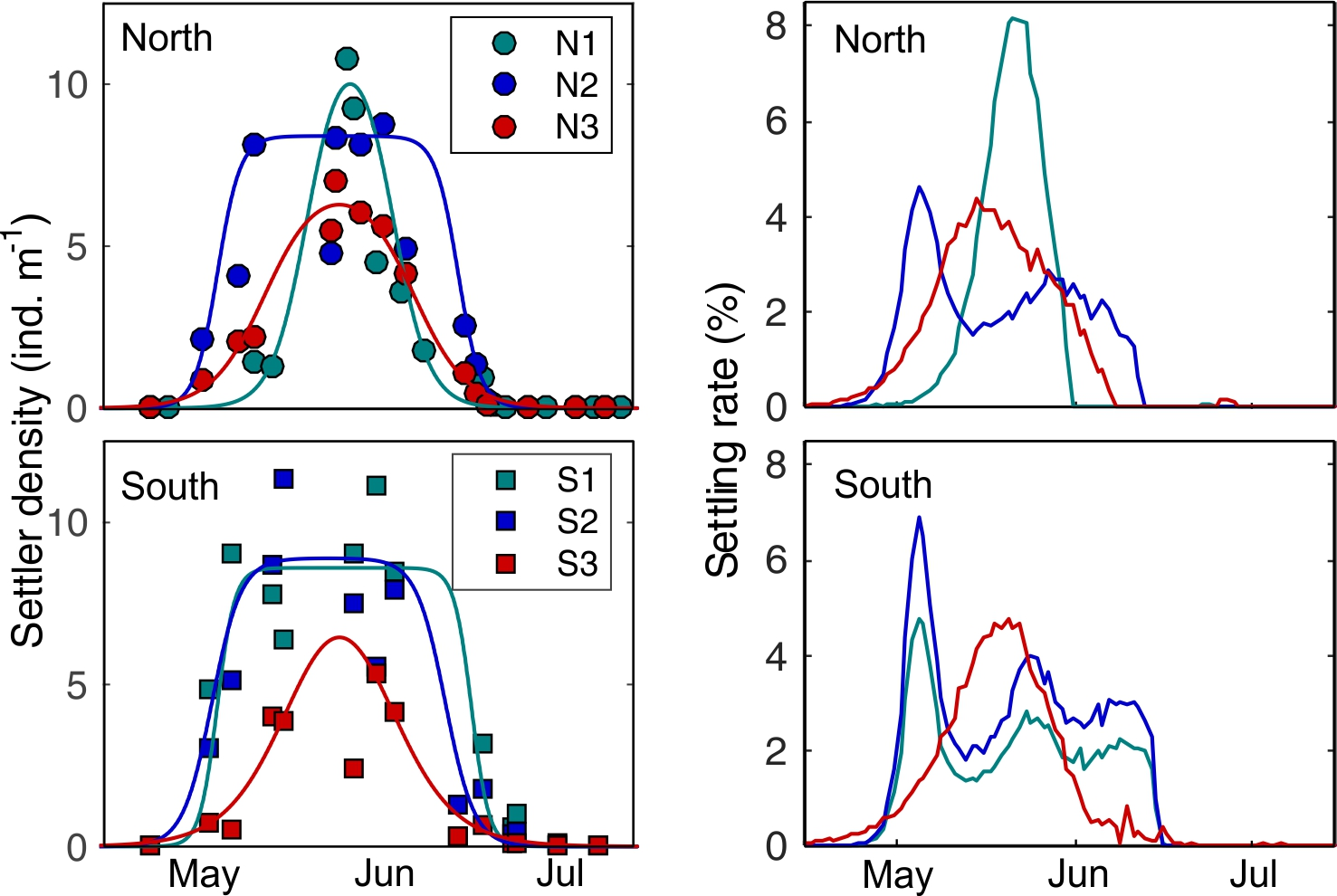

Supplement: S1 Fig — Left panels: measured settler (10–20 mm) density variation (dots and squares) and adjusted double sigmoid functions (lines). Right panels: values are indicated as daily % of total settlers arriving to each cove. (TIF) [file pone.0190278.s003.tif]

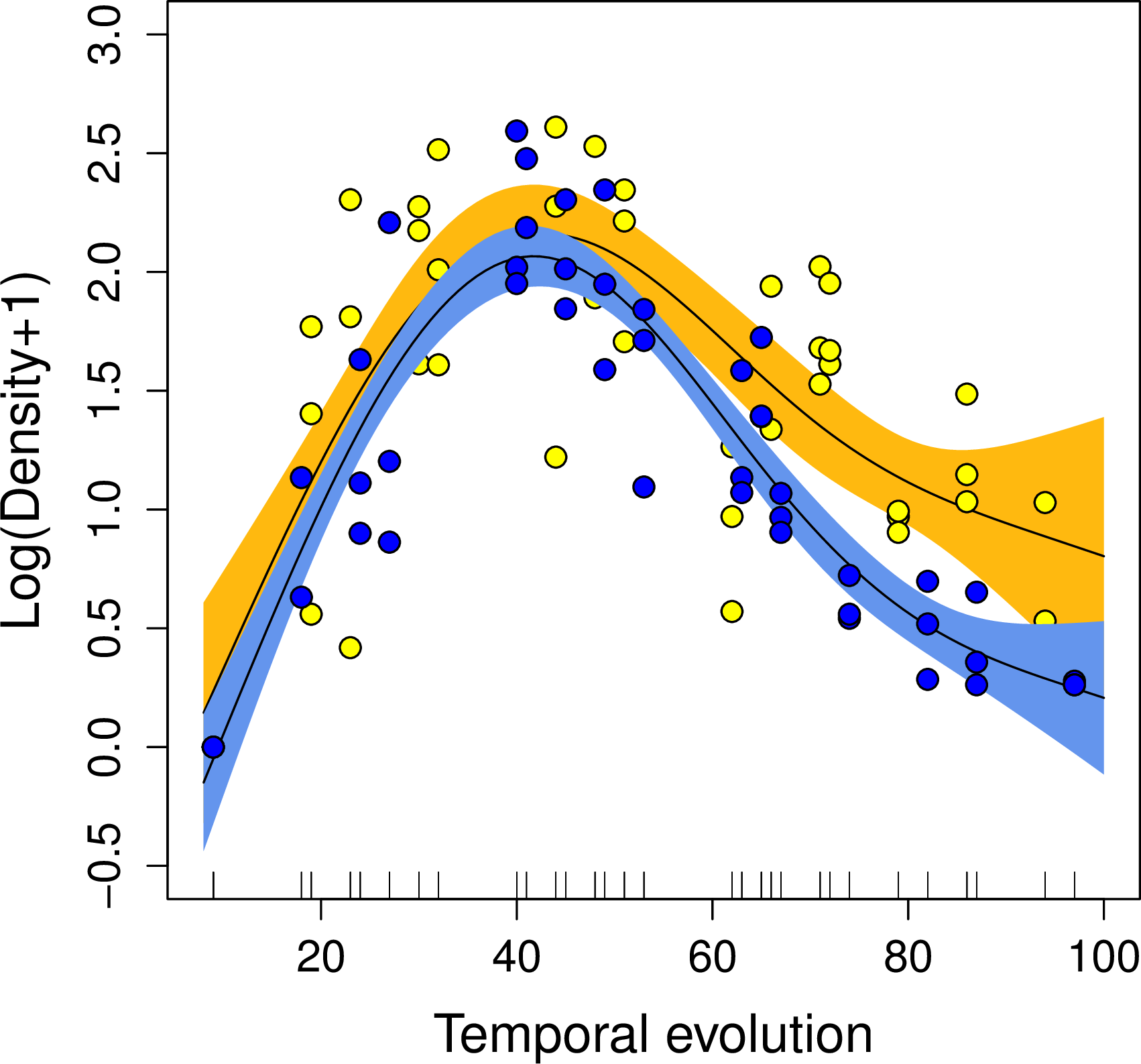

Supplement: S2 Fig — Blue represents the northeast coves, and yellow the southwest coves. Fitted lines (solid line), 95% confidence intervals (color shaded areas) and residuals (dots) are shown. (TIF) [file pone.0190278.s004.tif]

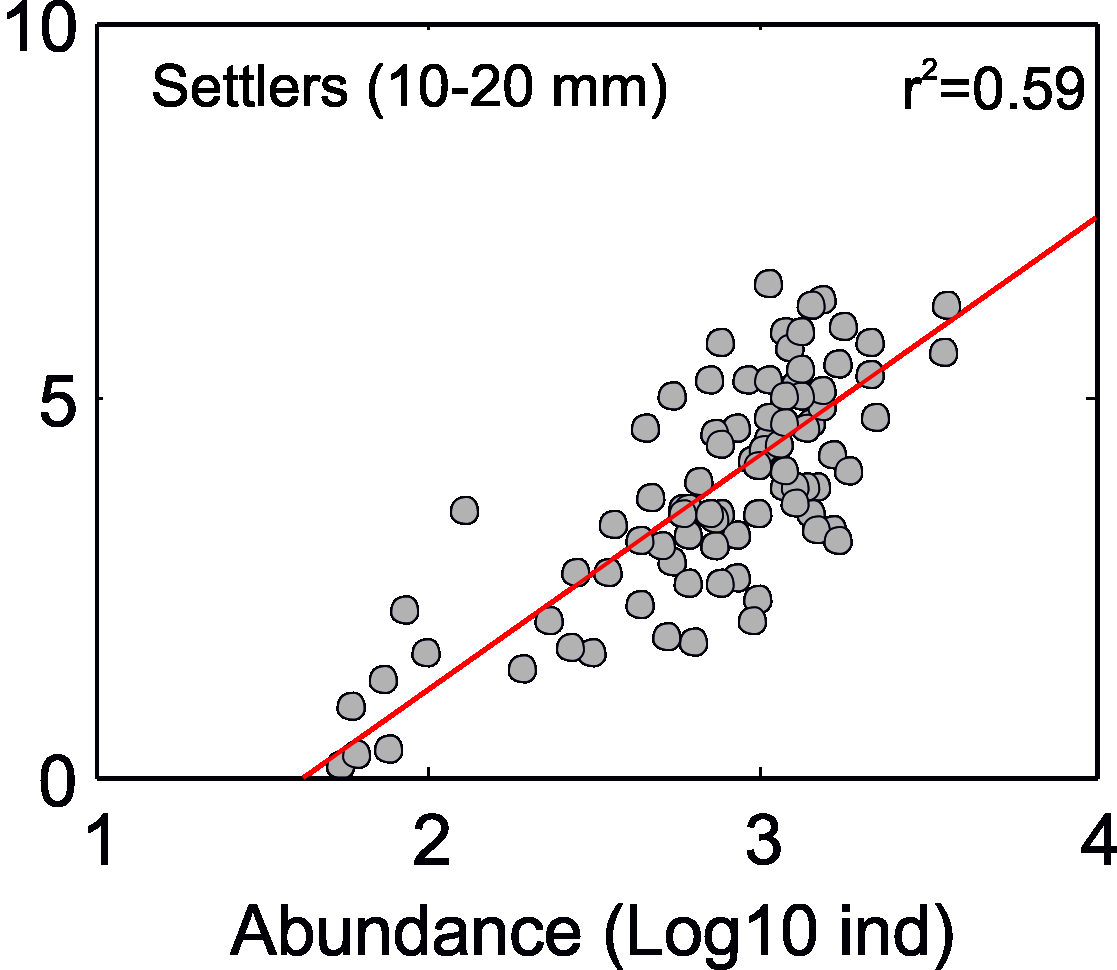

Supplement: S3 Fig — Density-dependent mortality relationship for juveniles smaller than 20 mm including all the individuals counted in the six coves. The vertical axis represents the daily mortality rate in percentage. (TIF) [file pone.0190278.s005.tif]
